# Why we need to depict the structure of DNA correctly: it’s so groovy

**DOI:** 10.1093/brain/awag097

**Published:** 2026-03-11

**Authors:** Elizabeth M C Fisher

**Affiliations:** Department of Neuromuscular Diseases and Queen Square Motor Neuron Disease Centre, UCL Queen Square Institute of Neurology, London WC1N 3BG, UK

## Abstract

The symbol for DNA is all around us, on advertising and in the news. Yet it is almost always drawn incorrectly. Elizabeth Fisher explains how it should be depicted, and why it is important that we get the famous double helix right.

***A picture paints a thousand words. Then once it becomes commonplace, it sometimes magically turns into a symbol, but symbols still need to be accurate.***

We spend our lives immersed in symbols that give us instant information. Whether male/female/trans symbols for the lavatories, a symbol such as our country’s flag, or the winged emblem of the rather beautiful Aston Martin car that you and I might like to own, we know immediately what the common symbols around us represent. This is also true for scientific communication, and increasingly, journals and funding bodies are asking for graphical abstracts in which we all might recognize the familiar object resembling a fried egg is a cell or that the juxtaposed triangular shapes form a synapse. Instant digestible information! How efficient and how marvellous that humans from anywhere, speaking any language, can understand these symbols.

However, although symbols become commonplace, so that we don’t really look at them yet instantly register what they mean, they still need to give us correct information, and sometimes the devil is in the detail. Take the example of DNA. People with no knowledge of DNA, genetics or science, speak earnestly about how cooking pavlovas, or cross-country skiing, ‘is in their DNA’, implying something fundamental that we all understand. It’s a sloppy phrase, it ignores the whole nature–nurture debate, but we all get it. As a geneticist I can’t even summon up a sigh of mild condescension, as I know that language is fluid and evolves, and I should be pleased that people have an idea of what DNA is.

Because DNA is now a ‘Thing’, its symbol is all around us, denoting ‘scientific’ and presumably therefore ‘reliable’. A brief glance around shows that my toothpaste comes with a double helix on the tube, that a new university merger in London with a medical component has a big double helix on one of its posters, and that a recent BBC news report on a new cancer therapy had a graphic that used the DNA symbol. This double helix symbol is so ubiquitous that a quick survey of internet images throws up hundreds of double helices for you to slap onto whatever product you’re trying to sell or talk you’re about to give.

And almost every single one of the images is incorrect. Furthermore, the double helix is drawn wrongly in an astonishing number of graphical abstracts in journals and scientific presentations (Fig. 1).

**Figure 1:**
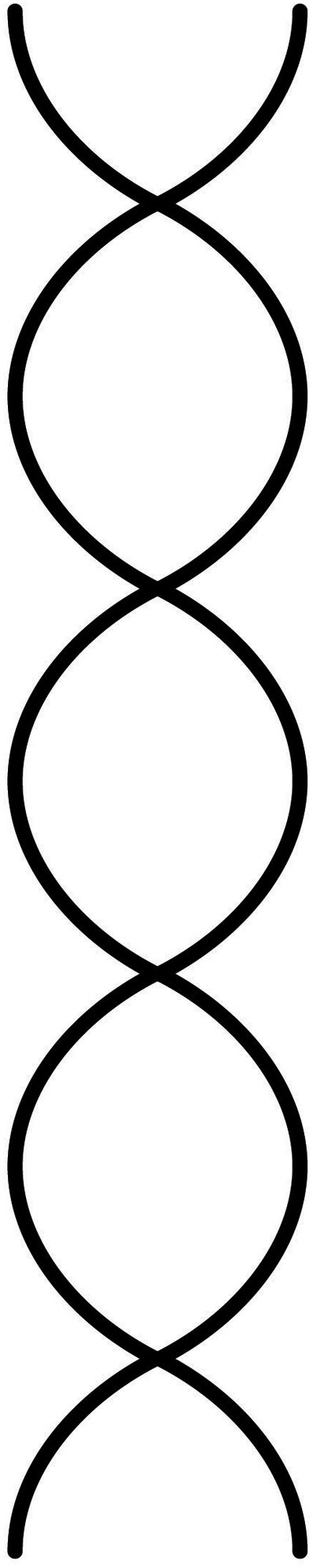
**Typical symbol representing a DNA double helix.** This symbol is perfectly symmetrical and represents approximately two turns of the helix. This symbol provides an incorrect representation of DNA.

It’s a simple but important issue. There are several forms of DNA but the usual structure that was made famous by Watson and Crick and Franklin and Wilkins is called the B form. The B form is by far the most predominant species in cells and therefore is the most important biologically active DNA structure. This is indeed a double helix, and the standard form which we all learn about in school. However, it is not a perfectly symmetrical molecule but is slightly offset, which crucially results in a major groove and a minor groove running the length of the molecule (Fig. 2).

**Figure 2:**
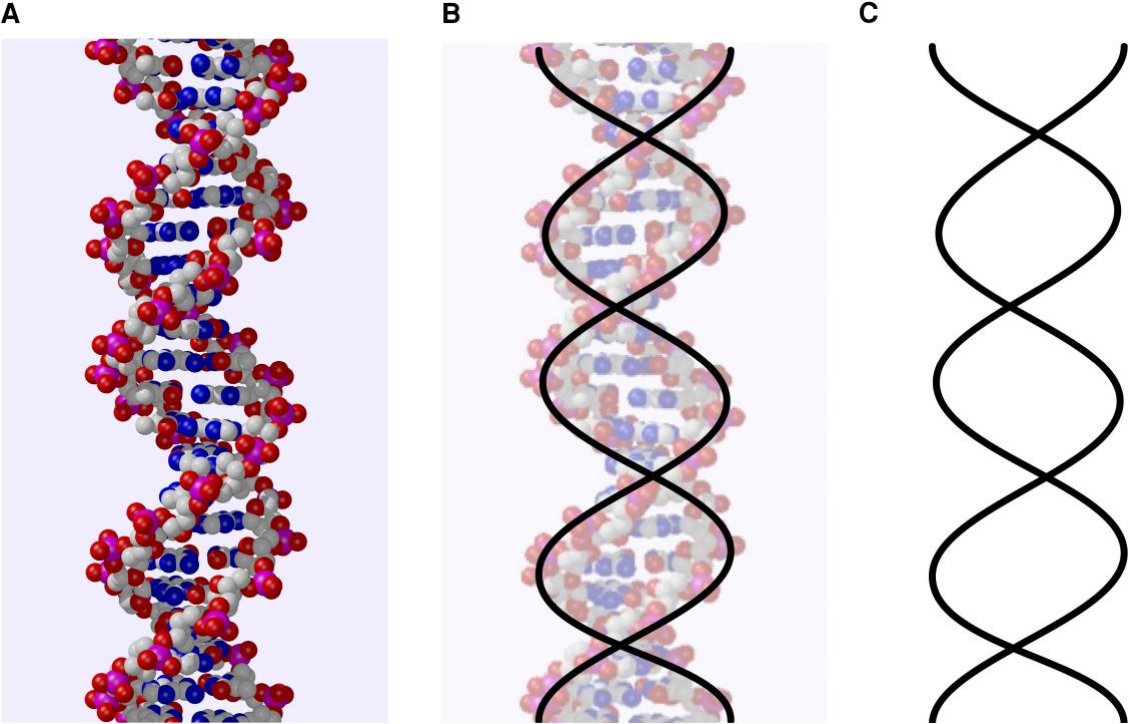
**Structure of the B form of DNA.** (**A**) A model structure of DNA, showing approximately two turns of the double helix. The major and the minor grooves are formed along the outside of the double helix. The red and white moieties are the alternating sugars and phosphates, covalently attached to each other, that make up each chain of the double helix. The ‘cross-links’ shown in blue and white are nucleotides, which are held together by hydrogen bonds. (**B**) The same model, with black lines running along the sugar-phosphate ‘backbone’ of each helix. (**C**) The same black lines, producing an accurate symbol structure of the B form DNA double helix. [Figure credit: (**A**) DNA Double Helix. Peter Artymiuk. Source: Wellcome Collection (dna | Images search | Wellcome Collection) used under a Creative Commons Licence CC BY 4.0 and modified with permission in **B** and **C**].

Why is this offset important? To answer this, we have to understand a bit about the structure and function of DNA, at least the B form. The DNA double helix consists of two polynucleotide chains that are ‘anti-parallel’ and twist around each other on the same axis. These two chains are held together in their double helix by hydrogen bonds between the ‘bases’ [adenine (A), thymine (T), cytosine (C), guanine (G)] that lie on each chain. Adenine and guanine are purines (consisting of double rings of atoms) and thymine and cytosine are pyrimidines (consisting of a single ring of atoms). In DNA, A and T pair together and G and C pair together, keeping the two nucleotide chains in their double helix.

The polynucleotide chains have directionality. This is because of their chemical composition: each chain has what is known as a 5′ end and a 3′ end—the 5′ and 3′ refer to specific carbon atoms that lie at opposite ends of the polynucleotide chains. The B form of DNA is an anti-parallel double helix, which simply means the two chains are wrapped around each and run in opposite directions.

Helices can have a right-handed or a left-handed spiral. Right-handed helices—such as the B form of DNA—turn away from you clockwise, if you look down at the helix from above; conversely, left-handed helices turn away anti-clockwise. Both chains have about 10.4 bases per turn, and the same twist or ‘pitch’ of ∼3.4 nm (i.e. one complete turn) or about 3.4 nm per ∼10 bases (depending on the chemical conditions in which the DNA is being studied).

Importantly—and crucially for its function but unlike most DNA symbols—DNA is not a perfectly symmetrical double helix. Because of their chemical composition, the two polynucleotide chains are slightly offset from each other. As a result, two grooves are formed that run along the outside of the molecule. There is a ‘major groove’ that is 2.2 nm wide and a ‘minor groove’ that is 1.2 nm wide. And these grooves are super important for how DNA works.

The major groove allows more access to the shapes of the underlying bases, so, for example, this is where DNA replication enzymes and the transcription factors responsible for gene expression tend to bind so that they can ‘read’ the DNA sequence. The minor groove can also contain transcription factors and has other binding sites, for example, for some histones, and small molecules, such as some drugs and water molecules. DNA binding proteins of all sorts have their individual preferences for the major or the minor grooves, based on how these proteins do their job. The grooves are essential for how DNA functions.

To be useful, symbols need to strike a balance. They need to be sufficiently simple to communicate effectively, but also sufficiently complex to convey the necessary information associated with them. Incorrect perfect helices that represent ‘DNA’ do not capture the asymmetry and essential grooved nature of the underlying molecule.

Does it matter that the double helix is incorrectly drawn in most DNA symbols? Or is this article just the ranting of an ageing professor? Clearly the answer to both questions is ‘yes’.

However, to explain, using the correct offset DNA symbol matters, first, because the perfect double helix symbol is widely used commercially to denote a sense of serious science, which is ironic given that the symmetrical helix is simply wrong. Second, for us academic types, we are supposed to be in pursuit of truth, not graphic design (although the best graphic design incorporates truth). Truth matters a lot to us because we spend our working lives trying to hunt it down. So we need to be careful about the symbols that we use to present the truth, rejecting the ones that are too simplified to do so effectively. There’s a parallel here with road signs. If you are driving in the UK what does a solid blue circle with a white number 30 on it mean? In surveys, ∼50% of people (including the author) didn’t know (it means the minimum legal speed is 30 miles per hour). In other words, the sign is sufficiently simple to read easily, but too stylized to convey the information it needs to impart. This matters all the more in our current media environment, where short-form communication that depends heavily on oversimplification is eliminating important nuance.

Finally, the incorrect and ubiquitous symmetrical double helix image helps promulgate a view, often proposed by the don’t-trust-modern-medicine brigade, that nature is perfect and therefore a perfect helix simply feels right. Nature isn’t perfect. Evolution wouldn’t occur if it were, and we need to embrace this fact. Nature is instead damn clever and makes use of imperfection and errors to create the wonderfully diverse world we live in.

DNA is simply incredible. Showing the offset in the DNA double helix is a reminder to all of us of the multitude of functions that it is involved in, and the many different proteins that need to bind to it to facilitate these, such as gene expression or gene regulation. DNA is an amazing molecule. Super-efficient, encoding vast amounts of data, uniquely personal for each of us, in the air long after you’ve left the room, massively long and twisty and dynamic, and changeable and yet stable over aeons. The structure enables replication, the encoding of genetic and epigenetic information, and protein synthesis. And we still don’t know what a lot of it does.

Let me leave you with the thought that there’s enough DNA in your body that end-to-end it could go to the moon and back up to 150 000 times. Got that? No, I can’t really imagine it either. So, let’s just get the DNA double helix symbol correct, in its many scientific and non-science appearances.

